# Quantitative assessment and clinical relevance of kallikrein-related peptidase 5 mRNA expression in advanced high-grade serous ovarian cancer

**DOI:** 10.1186/s12885-019-5901-0

**Published:** 2019-07-15

**Authors:** Weiwei Gong, Yueyang Liu, Christof Seidl, Eleftherios P. Diamandis, Marion Kiechle, Enken Drecoll, Matthias Kotzsch, Viktor Magdolen, Julia Dorn

**Affiliations:** 1 Clinical Research Unit, Department of Obstetrics and Gynecology, Technical University of Munich, Munich, Germany; 2 Division of Clinical Biochemistry, Department of Laboratory Medicine and Pathobiology, University of Toronto, Ontario, Canada; 3 Department of Institute of Pathology, Technical University of Munich, Munich, Germany; 4 Medizinisches Labor Ostsachsen, Dresden, Germany

**Keywords:** KLK5, Ovarian cancer, Quantitative PCR, mRNA expression

## Abstract

**Background:**

In ovarian cancer, dysregulation of mRNA expression of several components of the family of the kallikrein-related peptidases (KLKs) is observed. In this study, we have analyzed the KLK5 mRNA expression pattern in tumor tissue of patients suffering from high-grade serous ovarian cancer stage FIGO III/IV. Moreover, we have correlated the KLK5 mRNA levels with clinical outcome.

**Methods:**

We assessed the mRNA expression levels of KLK5 in tumor tissue of 138 patients using quantitative PCR (qPCR). The mRNA levels were correlated with KLK5 antigen tumor tissue levels measured by ELISA (available for 41 of the 138 patients), established clinical features as well as patients’ outcome, using Chi-square-tests, Mann-Whitney U-tests and Spearman rank calculations as well as Cox regression models, Kaplan-Meier survival analysis and the log-rank test.

**Results:**

A highly significant correlation between the mRNA expression levels and protein levels of KLK5 in tumor tissues was observed (r_s_ = 0.683, *p* < 0.001). In univariate Cox regression analysis, elevated KLK5 mRNA expression was remarkably associated with reduced progression-free survival (PFS; *p* = 0.047), but not with overall survival (OS). Association of KLK5 mRNA expression with PFS was validated in silico using The Cancer Genome Atlas. For this, Affymetrix-based mRNA data (*n* = 377) were analyzed applying the Kaplan-Meier Plotter tool (*p* = 0.027). In multivariable Cox analysis, KLK5 mRNA values revealed a trend towards statistical significance for PFS (*p* = 0.095), whereas residual tumor mass (0 mm vs. > 0 mm), but not ascites fluid volume (≤500 ml vs. > 500 ml), remained an independent indicator for both OS and PFS (*p* < 0.001, *p* = 0.005, respectively).

**Conclusions:**

These results obtained with a homogenous patient group with all patients suffering from advanced high-grade serous ovarian cancer support previous results suggesting elevated KLK5 mRNA levels as an unfavorable marker in ovarian cancer.

## Background

In women, ovarian cancer (OC) is the fifth common cause of cancer-related death [1]. In 2018, over 22,000 new OC cases and about 14,000 deaths due to OC are estimated [2]. Because 75% of OCs are detected at late stage (FIGO stage III and IV) [3], the 5-year survival of patients is less than 47.4% [2]. Late detection of OC is due to the fact that early stage OC frequently does not cause any obvious or specific symptoms [4]. Hence, novel tumor biomarkers for early detection, diagnosis and prognosis of OC are strongly required.

The human kallikrein-related peptidase gene family (*KLK1–15*) is located within a single cluster in the chromosomal region 19q13.4. All KLK peptidases belong to the chymotrypsin (S1) family of secreted serine proteases [5]. In the past years, numerous reports indicated that KLKs are aberrantly expressed in malignancies of the breast, ovary, prostate, bladder, colon, stomach, lung, and brain [6–8]. Moreover, accumulating reports demonstrated that KLKs, when overexpressed in malignant tissues, are also detectable in serum and in effusion fluids, consistent with the fact that KLKs are secreted by epithelial/glandular cells [9, 10]. Thus, KLKs may serve as biomarkers in detection of primary cancer, clinical diagnosis, and prediction of the clinical outcome [5].

KLK5 was originally identified as the stratum corneum tryptic enzyme in the normal human epidermis, where it is involved in degradation of corneodesmosomes in the outermost layer of human skin, resulting in skin desquamation [11]. Previous studies have shown that KLK5 mRNA and protein are differentially expressed especially in prostate, ovarian, breast, and testicular tumors [12–17]. In previous OC-related studies it has been described that KLK5 expression is correlated with progressive disease as well as higher tumor grade [12, 18]. Moreover, overexpression of KLK5 at mRNA and protein level was found to be associated with poor prognosis of OC patients both with respect to progression-free (PFS) and overall survival (OS) [19, 20]. Using OVMZ-6 OC cells simultaneously overexpressing KLK4–7, we observed that KLK5 in concert with the other three KLKs leads in vitro to an induction of TGFß-1 signaling, increased invasion, and chemoresistance as well as enhanced tumor burden in vivo [21–24].

It is noteworthy, that in most of the previous OC-related studies the patient cohorts encompassed low- and high-grade tumors as well as different histological types (serous, mucinous, endometroid, clear cell) of OC (see e.g. [12, 19]). Hence, to further specify the clinical value of KLK5, we have now analyzed KLK5 mRNA expression and its association with patients’ outcome in a defined OC subgroup, advanced high-grade serous ovarian cancer (HGSOC).

## Methods

### Patients

A total of 138 different specimens of primary tumor tissues from patients with advanced high-grade serous ovarian cancer (HGSOC): FIGO IIIA (*n* = 5), IIIB (*n* = 10), IIIC (*n* = 92), and IV (*n* = 31) diagnosed between 1990 and 2012 were enrolled in the study. Cases were identified upon availability of clinical data and tumor specimens. The samples were retrieved from the biobank of the Department of Obstetrics and Gynecology and the Institute of Pathology (which is part of the biobank of the Klinikum rechts der Isar, TU Munich, Germany). After surgical resection of the primary tumor, tissue samples were examined by pathologists of the Institute of Pathology, TU Munich, representative areas of the tumor tissue selected, immediately snap-frozen, and then stored in liquid nitrogen. Purified RNA (and cDNA) was preserved at − 80 °C. All patients received standard stage-related primary radical debulking surgery at the Department of Obstetrics and Gynecology, Klinikum rechts der Isar, TU Munich. For 70 of the patients, no residual tumor mass was visible after surgery.

No chemotherapy was applied before primary surgery. All patients received systemic adjuvant treatment after surgery, including platinum-based chemotherapy, according to therapy standards at time of diagnosis. Clinical data and follow-up information were collected, first, by including the patients in our in-house database of ovarian cancer patients after surgery; second, during follow-up visits; and, third, retrospectively using patient’s files and the Munich tumor registry. Taken together, we obtained high quality data and, for some patients, very long follow up times. Still, because in the tumor registries sometimes only data concerning OS time are available, information about PFS can be missing, giving for some patients not a complete information about the course of the disease. In 12 of the 138 cases for OS and 30 cases for PFS, no follow-up data could be retrieved. Furthermore, for survival analysis, cases with an event occurring earlier than ≤3 months (7 cases for OS and 3 cases for PFS) were excluded. All in all, 119 patients could be included in OS, 105 patients in PFS analyses. Follow-up information was available in the range of ≥4 to ≤279 months for both OS (median time, 31 months) and PFS (median time, 20 months).

### KLK5 antigen levels

The KLK5 antigen levels of 41 of the 138 cases of the present patient cohort have been determined by an immunofluorometric assay (ELISA) in previous studies [19, 25]. The ELISA determinations and the qPCR analyses of the present paper were performed with independent tissue samples of the same patient.

### Quantitative real-time PCR

A detailed description of total RNA extraction, reverse transcription of the mRNA, first-strand cDNA synthesis, and quantitative real-time polymerase chain reaction (qPCR) has been previously published [26]. The qPCR assay for quantification of KLK5 mRNA expression was established in-house applying the Universal ProbeLibrary Assay Design Center software and the Universal ProbeLibrary (Roche, Penzberg, Germany). HPRT1 was used as reference gene [26].

The primers (5′-AAGGCCCAACCAGCTCTACT-3′ and 3′-CCGAGACGGACTCTGAAAAC-5′) were specific for KLK5. 5′-FAM-GCAGGAAG (Universal Probe Library) was used as hydrolysis probe. Three major transcript variants of KLK5 mRNA are detected in the KLK5 qPCR assay (NM_001077491.1, variant 1; NM_001077492.1, variant 2; NM_012427.4, variant 3), all encoding full length protein.

Standard dilution series have been utilized for substantiating the reaction efficiency and sensitivity, particularly considering slightly divergent process steps of RNA extractions and reverse transcriptions performed at different time points as well as different lot numbers of the master mix [27]. The 2exp-ΔΔCt method was used for relative quantification [28].

### Statistical analysis

Analyses were performed using the SPSS statistical analysis software (version 20.0; SPSS Inc., Chicago, IL, USA) (for details see [26]). In all statistical tests of this study, differences were considered to be significant if the *p* value was < 0.05.

## Results

### Quantification of KLK5 mRNA levels in tumor tissue samples of advanced high-grade serous ovarian cancer patients (FIGO III/IV)

**Fig. 1:**
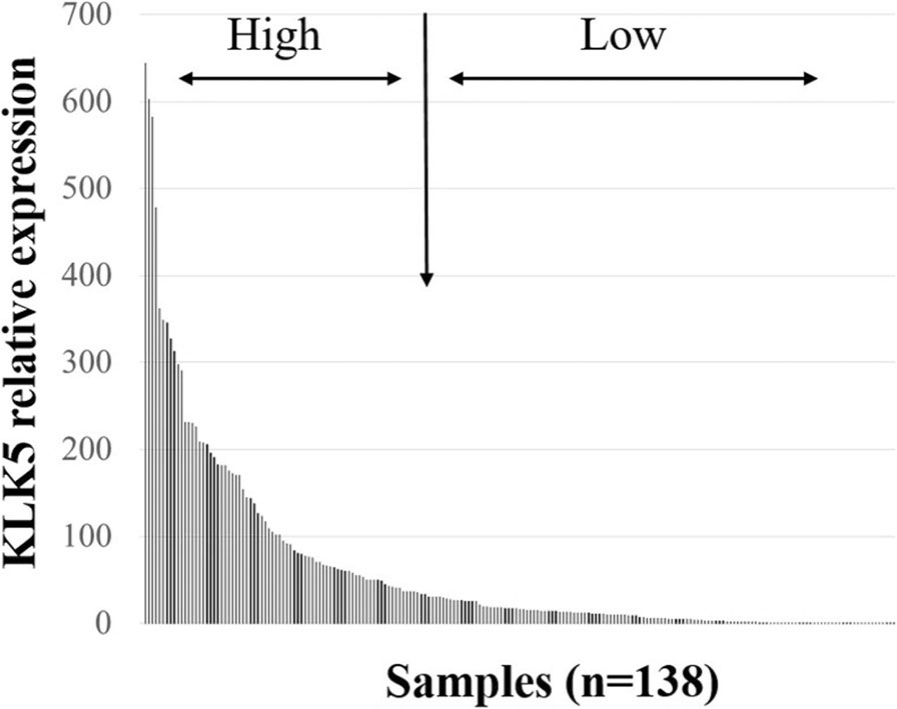
KLK5 mRNA expression in tumor tissue samples of patients suffering from advanced HGSOC stage FIGO III/IV. The histogram depicts expression of KLK5 mRNA relative to the HPRT1 mRNA expression. For further analysis, we categorized the KLK5 mRNA expression levels by the lower two tertiles (T1 + 2) in a low expression group versus a group showing high expression encompassing the highest tertile (T3)

**Fig. 2:**
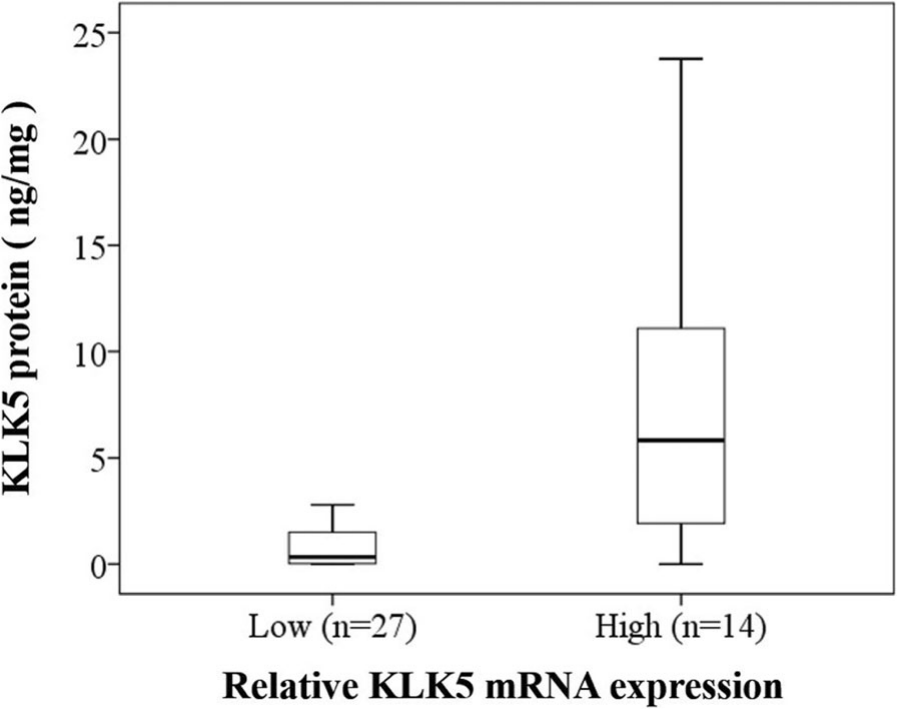
Correlation between mRNA expression levels and protein levels of KLK5 in tumor tissues of patients. A highly significant correlation is observed between the mRNA expression levels (determined by qPCR) and protein levels of KLK5 (measured by ELISA in cytosolic extracts of ovarian cancer patients’ tumor tissues). Mann Whitney U-test, *p* < 0.001

KLK5 mRNA expression was quantified by qPCR in tissue samples of 138 patients. At time of surgery, the patients were at least 33 and at most 88 years old (median: 64 years). The relative KLK5 mRNA levels ranged from 0 to 644.31 (median = 16.87). The KLK5 mRNA expression levels were classified by the lower two tertiles (T1 + 2) in the low expression group versus the highest tertile (T3) representing the high expression group (Fig. 1). In 41 of the 138 cases, KLK5 antigen levels - determined in cytosolic tumor tissue extracts by ELISA - were available. Here, we observed a highly significant correlation between KLK5 mRNA expression and protein levels both by applying the Mann-Whitney U-test (*p* < 0.001; Fig. 2) and the Spearman rank correlation (r_s_ = 0.683, *p* < 0.001). Table 1 shows the correlations of KLK5 mRNA expression levels with clinical parameters (age [≤ 60 years vs. > 60 years], post-operative residual tumor mass [0 mm vs. > 0 mm], and pre-operative ascites fluid volume [≤ 500 ml vs. > 500 ml]), showing a significant association between KLK5 mRNA expression and residual tumor mass (*p* = 0.041) and a trend towards significance between KLK5 mRNA expression and the FIGO stage (*p* = 0.051).

**Table 1:** Association between KLK5 mRNA expression levels and clinical characteristics in patients with advanced HGSOC stage FIGO III/IV

Clinical parameters	No. of patients	KLK5
		Low/high ^a^
Age		*p* = 0.476
≤ 60 years	58	35 (60%)/23 (40%)
> 60 years	80	53 (66%)/27 (34%)
Residual tumor mass		***p*** **= 0.041**
0 mm	70	51 (73%)/19 (27%)
> 0 mm	66	37 (56%)/29 (44%)
Ascites fluid volume		*p* = 0.087
≤ 500 ml	77	54 (70%)/23 (30%)
> 500 ml	54	30 (56%)/24 (44%)
FIGO stage		*p* = 0.051
IIIA + IIIB	15	13 (87%)/2 (13%)
IIIC + IV	123	75 (61%)/48 (39%)

Due to missing values, numbers do not always add up to *n* = 138

^a^ Categorized into low- and high-expressing groups by tertiles 1 + 2 versus tertile 3

Significant *p*-value is indicated in bold

### Association of clinical parameters as well as KLK5 mRNA expression levels with patients’ survival

**Fig. 3:**
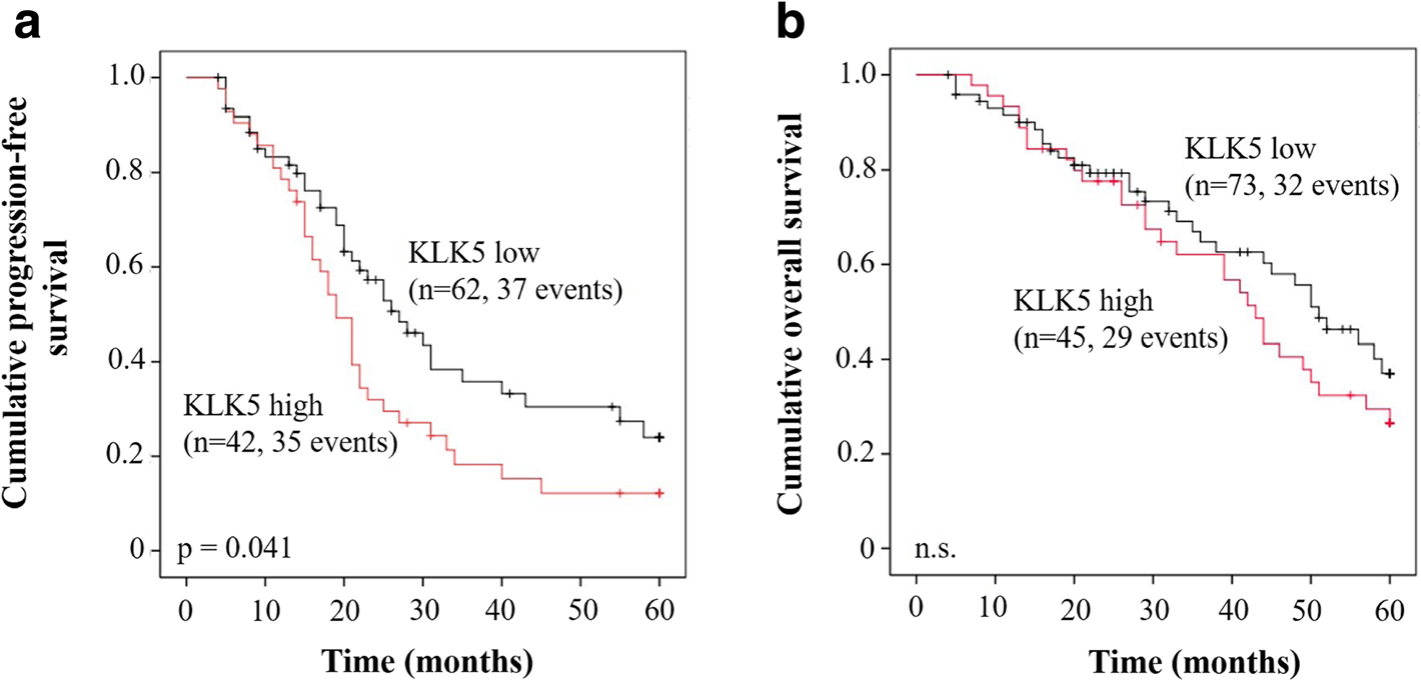
Probability of PFS and OS in correlation with expression of KLK5 mRNA in primary tumor tissue samples of patients afflicted with advanced HGSOC stage FIGO III/IV. Patients showing high expression of KLK5 mRNA display a significantly worse PFS (Kaplan-Meier analysis, *p* = 0.041) (**a**) but not OS (**b**), compared to patients with low KLK5 mRNA expression levels

Table 2 summarizes the results of univariate Cox regression analysis demonstrating an association of established clinical parameters as well as of KLK5 mRNA expression with both OS and PFS of the patients (observation time: 5 years). OS data were available for 119 patients, PFS data for 105 patients. The clinical parameters residual tumor mass (0 mm vs. > 0 mm) and ascites fluid volume (≤ 500 ml vs. > 500 ml) proved to be univariate predictors for both OS and PFS, whereas the clinical parameter age was neither significantly associated with OS nor with PFS. Elevated mRNA expression levels of KLK5 were shown to be markedly correlated with poor PFS (hazard ratio [HR] = 1.60, 95% CI = 1.01–2.55, *p* = 0.047). However, with regard to OS, no significant correlation with KLK5 mRNA expression levels was observed (Table 2). Kaplan-Meier survival curves confirm these results. Here, a significant difference between high and low KLK5 expression concerning PFS (*p* = 0.041) but not OS (Fig. 3) can be seen as well.

**Table 2:** Univariate Cox regression analysis of KLK5 mRNA expression levels and patients’ survival in advanced HGSOC stage FIGO III/IV

Clinical parameters	OS			PFS		
	No ^a^	HR (95% CI) ^b^	p	No ^a^	HR (95% CI) ^b^	p
Age			0.414			0.762
≤ 60 years	49	1		43	1	
> 60 years	70	1.24 (0.74–2.06)		62	1.08 (0.67–1.72)	
Residual tumor mass			**< 0.001**			**< 0.001**
0 mm	60	1		58	1	
> 0 mm	57	3.80 (2.17–6.65)		47	2.41 (1.53–3.90)	
Ascites fluid volume			**0.005**			**0.019**
≤ 500 ml	66	1		61	1	
> 500 ml	46	2.10 (1.25–3.54)		38	1.79 (1.10–2.90)	
FIGO stage			0.215			0.360
IIIA + IIIB	13	1		12	1	
IIIC + IV	106	1.90 (0.69–5.24)		93	1.44 (0.63–3.14)	
KLK5 mRNA ^c^			0.269			**0.047**
low	73	1		62	1	
high	45	1.33 (0.80–2.20)		42	1.60 (1.01–2.55)	

Significant *p*-values (*p* < 0.05) are indicated in bold. Due to missing values, numbers do not always add up to *n* = 119 (OS) and *n* = 105 (PFS)

^a^ Number of patients;

^b^ HR: hazard ratio (CI: confidence interval) of univariate Cox regression analysis;

^c^ Dichotomized into low and high levels by tertiles 1 + 2 versus tertile 3;

**Fig. 4:**
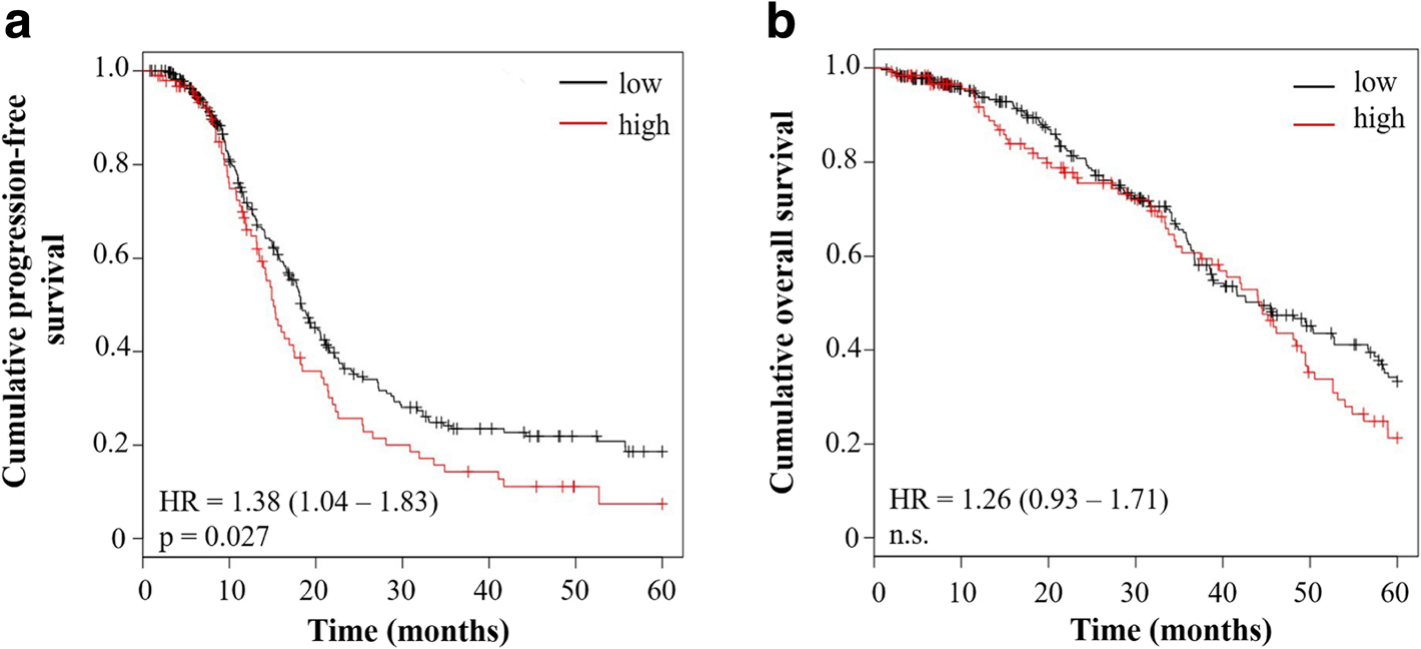
Validation of a significant association between expression of KLK5 mRNA and survival of patients using accessible Affymetrix microarray data. For analysis of the prognostic value of KLK5 expression, the microarray data provided by ‘The Cancer Genome Atlas (TCGA)’ (probe ID 222242_s_at) were used. Selection criteria for patients included (i) serous histological type, (ii) advanced stage (FIGO III/IV), (iii) high-grade (grade 3), (iv) chemotherapy using platinum compounds, and (v) a follow-up of 5 years. The selection resulted in a patient cohort encompassing 377 patients for analysis of the association with PFS (**a**) and 398 patients for PFS (**b**), respectively

To validate these results, we used Affymetrix-based mRNA data provided by ‘The Cancer Genome Atlas (TCGA)’ and the program Kaplan-Meier Plotter [29]. For this in silico analysis, we selected for patients suffering from HGSOC in advanced stage (FIGO III + IV) treated with a platinum-based chemotherapy, resulting in patient cohorts with 398 patients for OS and 377 patients for PFS analysis, respectively. In line with our results presented in Fig. 3, Kaplan-Meier analysis (with 5 years follow up) confirmed that, in these OC cohorts, elevated KLK5 mRNA levels showed a significant correlation with a shortened PFS (*p* = 0.027) but again not with OS (Fig. 4).

Next, a multivariable analysis was performed to further evaluate the impact of KLK5 mRNA expression on prognosis. The base model included the clinical parameters age, residual tumor mass, and ascites fluid volume to which the parameter KLK5 mRNA expression was subsequently added (Table 3).

**Table 3:** Multivariable Cox regression analysis of KLK5 mRNA expression levels and patients survival in advanced HGSOC stage FIGO III/IV

Clinical parameters	OS			PFS		
	No ^a^	HR (95% CI) ^b^	p	No ^a^	HR (95% CI) ^b^	p
Age			0.660			0.592
≤ 60 years	46	1		41	1	
> 60 years	63	1.13 (0.65–1.96)		57	0.87 (0.53–1.44)	
Residual tumor mass			**< 0.001**			**0.005**
0 mm	59	1		57	1	
> 0 mm	50	3.29 (1.69–6.41)		41	2.20 (1.17–3.81)	
Ascitic fluid volume			0.605			0.363
≤ 500 ml	64	1		60	1	
> 500 ml	45	1.18 (0.64–1.91)		38	1.33 (0.76–2.31)	
KLK5 mRNA ^c^			0.718			0.095
low	69	1		59	1	
high	40	1.11 (0.64–1.91)		39	1.53 (0.93–2.51)	

The biological marker KLK5 mRNA was added to the base model of clinical parameters: age, residual tumor mass, and ascites fluid volume. Significant *p*-values (*p* < 0.05) are indicated in bold

^a^ Number of patients;

^b^ HR: hazard ratio (CI: confidence interval) of multivariable Cox regression analysis;

^c^ Dichotomized into low and high levels by tertiles 1 + 2 versus tertile 3

In the base model, only residual tumor mass remained as an independent indicator both for OS (HR = 3.29, 95% CI = 1.69–6.41, *p* < 0.001) and PFS (HR = 2.20, 95% CI = 1.27–3.81, *p* = 0.005). Upon addition to the base model, KLK5 mRNA expression did not prove to be statistically significant, however, showed a tendency with regard to statistical significance in case of PFS (HR = 1.53, 95% Cl = 0.93–2.51, *p* = 0.095). In contrast, the clinical parameter residual tumor mass represented a statistically significant, independent factor for OS as well as PFS.

## Discussion

So far, several studies have been published dealing with the prognostic relevance of KLK5 in OC. In a PCR-based study, Kim and co-workers [12] reported that higher KLK5 mRNA levels are associated not only with advanced stage and higher grade, but also with failure of response to chemotherapy. Furthermore, higher KLK5 mRNA levels were linked to unfavorable PFS and OS. Similar results were observed when KLK5 antigen levels, determined by ELISA, were compared in extracts of epithelial ovarian tumor tissue and of low malignant potential (LMP) tumors. There, KLK5 antigen levels were significantly higher in ovarian tumor tissue than in LMP tumors [19]. Furthermore, elevated KLK5 levels were associated with later stage and higher grade as well as shorter PFS and OS. In another ELISA-based study, high KLK5 levels were linked to unfavorable PFS in univariate analysis [30]. Interestingly, larger KLK5 level differentials (between primary tumor and omentum metastasis), as measured by ELISA, were also associated with shorter PFS [31], which may indicate a tumor-supporting role of KLK5 also during metastasis and not only in primary tumor growth. Together with KLK6 and KLK7, KLK5 belongs to the highly expressed KLK genes in OC [32, 33]. Therefore, it is not surprising that KLK5 antigen can also be measured in body fluids (serum; ascites fluid) of patients suffering from OC. In fact, higher KLK5 protein levels were measured in serum samples of patients afflicted with OC compared to healthy controls, benign and/or LMP tumors [20, 34]. Furthermore, elevated KLK5 antigen levels were observed in advanced versus early stages of OC [34]. In both, uni- and multivariate analyses, elevated KLK5 serum levels have been linked to both shorter PFS and OS [35], while in another study a statistically significant association between high KLK5 serum levels with shorter PFS was found in univariate analysis [20]. Taken together, there are data indicating an association between increased KLK5 expression in OC patients and poor prognosis. In fact, there is only one study pointing to a tumor-suppressive function of KLK5 in tumor tissue. There, expression of KLK5 was analyzed by immunohistochemistry in tissue samples of patients suffering from advanced OC. Interestingly, an increased expression of KLK5 in tumor-associated stromal cells, but not in tumor cells, was found to be related to a favorable clinical outcome [25].

In most of the above described clinical studies, OC patients belonging to several subtypes were included, like high and low grade serous, mucinous, clear cell and endometrioid ovarian cancer. However, these subtypes are very different with regard to (i) the origin of the tumors, (ii) molecular characteristics, and (iii) course of the disease. [1, 36]. In the study presented here, we analyzed the clinical relevance of KLK5 mRNA expression in a cohort including exclusively patients suffering from high-grade serous ovarian cancer (HGSOC) stage FIGO III/IV. This histological subtype represents about 70% of all OC cases. It should be noted, however, that excluding all other OC subtypes does result in a rather small cohort size (*n* = 138).

In line with previous findings that enhanced KLK5 expression is correlated to a higher grade and advanced stage in OC patients, we observed robust KLK5 mRNA expression in the majority of the analyzed cases. Furthermore, mRNA levels were found to correlate with KLK5 protein antigen levels (r_s_ = 0.683, *p* < 0.001) indicating that in case of KLK5 expression there is no major post-transcriptional regulation and, thus, mRNA and protein data are comparable.

Our investigation of possible associations between KLK5 mRNA expression and clinical pathological parameters demonstrated that in advanced HGSOC there is a significant association between elevated KLK5 mRNA expression and residual tumor mass after surgery. A significantly increased proportion of tumors displaying elevated KLK5 mRNA expression (*p* = 0.041) was observed in the patient group with post-operative residual tumor (44%, 29/66), compared to the tumor-free group (27%, 19/70). This suggests an unfavorable role of KLK5 expression with respect to therapy success and therefore prognosis of OC patients. Indeed, in the study presented here, we could demonstrate that an elevated expression of KLK5 mRNA is significantly associated with shortened progression-free survival in univariate analysis (*p* = 0.047). This result could be confirmed by analysis of Affymetrix-based mRNA data gathered from patients suffering from HGSOC and made accessible for the research community [29]. In multivariable analysis, KLK5 mRNA expression lost significance (*p* = 0.095), which may be either due to the relative low numbers of included cases or its association with the strong clinical factor ‘residual tumor mass’, which remains as an independent factor (*p* < 0.001). Interestingly, in the study by Kim and co-workers [12], KLK5 mRNA expression, although significantly associated with both PFS and OS in univariate analysis, only showed independent prognostic value in the subset of tumors with lower grade disease (grades I and II), but not in high-grade tumors.

Neither in our patient cohort nor in the publicly available data set, we observed a statistically significant association of KLK5 mRNA expression with OS. This is in line with several other reports [20, 30, 31], which observed a statistically significant association of KLK5 expression with PFS only. However, other groups have reported that KLK5 expression is linked to both PFS and OS [12, 19]. Whether these differences are due to the rather varying composition of the different cohorts concerning histological subtypes and low-grade versus high-grade tumors can presently not be answered.

Under physiological conditions, one of the major functions of KLK5 - together with KLK7 - is to mediate turnover and desquamation of the skin via degradation of cell-cell and cell-matrix adhesion molecules. Thus, KLK5 overexpression might contribute to unfavorable prognosis in OC patients via supporting tumor cell shedding from the primary tumor as well as cleavage of extracellular matrix proteins during metastasis. In fact, KLK5 efficiently degrades a variety of extracellular matrix (ECM) proteins including fibronectin, laminin and collagen I, II, III and IV [37]. Furthermore, in breast cancer cells, KLK5 overexpression was shown to result in down-regulation of a multitude of miRNAs and up-regulation of another set of mRNAs, finally affecting miRNA networks involved in post-transcriptional gene regulation of ECM molecules and cell adhesion pathways [38]. Thus, the link between increased KLK5 levels with worse outcome indicates KLK5 to be a potential target for therapy.

Moreover, KLK5 targets a broad range of substrates such as insulin-like growth factor binding proteins, transforming growth factor (TGF-β), and protease-activated receptors (PAR), which upon (in-)activation by KLK5 modulate important tumor-associated signaling pathways [39, 40]. In a secretome and degradome profiling study, co-overexpression of KLK4–7 resulted in distinct more than two-fold changes in relative protein abundances as compared to KLK4–7-deficient ovarian cancer cells. Many of the identified differentially expressed proteins are involved in cell-cell communication, including TGF-ß [24]. Furthermore, KLK4–7 regulate gene expression of other cancer-related factors: simultaneous overexpression of these four KLKs lead, e.g., to a distinct up-regulation of moesin and keratin 19 mRNA and protein expression, while keratin 7 is strongly down-regulated [41]. In fact, under physiological conditions, several members of the KLK family are known to interact with each other in different tissues [42]. Analysis of co-over- and under-expression of KLKs in OC and the impact on prognosis should be undertaken in the future, as it may shed more light on the pathobiology of the KLK network in OC. KLK5 may also affect the extracellular proteolytic network in the tumor cell microenvironment by activating the zymogen forms of other tumor-associated proteases including pro-urokinase-type plasminogen activator (pro-uPA) and pro-KLK11 [43]. It is of note that in other cancer cell types functional analyses have demonstrated an association of KLK5 with invasiveness and cell-cell cohesion. In bladder carcinoma cells, siRNA-mediated inhibition of KLK5 expression led to a significant reduction of invasion in Matrigel-based assays [44], whereas in oral squamous cell carcinoma cells silencing of KLK5 enforced cell-cell adhesion that promoted loss of junctional integrity and, hence, metastasis [45].

## Conclusions

In summary, the study presented here demonstrates that, in univariate analysis, elevated expression of KLK5 mRNA is significantly related with shortened PFS of patients afflicted with advanced HGSOC. This association was subsequently validated in silico using data from The Cancer Genome Atlas. These results, thus, indicate that KLK5 expression can be considered a prognostic biomarker for PFS in advanced HGSOC. Together with previous findings of clinical, biochemical, and cell biological studies pointing to a tumor-supporting role of KLK5, this kallikrein-related peptidase may be regarded as a novel target for therapy of OC and presumably of other cancers as well.

## Data Availability

Data are available via the Ethics Committee of the Medical Faculty of the Technical University of Munich, Ismaninger Str. 22, 81675 Munich, Germany, for researchers who meet the criteria for access to confidential data. According to the Bavarian Data Protection Authority (BayLDA) and the General Data Protection Regulation (GDPR), patient-related data will only be made available to third parties after double-pseudonymization, undertaken by the Dept. of Medical Statistics and Epidemiology, Technical University of Munich. The Ethics Committee of the Medical Faculty of the Technical University of Munich can be contacted at ethikkommission@mri.tum.de.

## Abbreviations

95% CI: 95% confidence interval
ECM: Extracellular matrix
HR: Hazard ratio
KLK: Kallikrein-related peptidase
OS: overall survival
PAR: Protease-activated receptor
PFS: Progression-free survival
qPCR: Quantitative PCR
TCGA: The Cancer Genome Atlas
TGF: Transforming growth factor
uPA: Urokinase-type plasminogen activator
